# The influence of temperature and humidity on the flight activity of *Culicoides imicola* both under laboratory and field conditions

**DOI:** 10.1186/s13071-018-3272-z

**Published:** 2019-01-03

**Authors:** Gert J. Venter, Solomon N. B. Boikanyo, Chantel J. de Beer

**Affiliations:** 1 Epidemiology, Parasites and Vectors, Agricultural Research Council-Onderstepoort Veterinary Research, Pretoria, South Africa; 2 Department of Veterinary Tropical Diseases, University of Pretoria, Pretoria, South Africa

**Keywords:** Host-seeking activity, Light trap efficiency, Risk

## Abstract

**Background:**

Insight into the factors that regulate the circadian host-seeking flight activity of *Culicoides* vectors (Diptera: Ceratopogonidae) will be of importance to assess the risk of transmission of *Culicoides*-borne pathogens. This study aimed to determine the impact of temperature and humidity on the flight activity of *Culicoides imicola* Kieffer, and other livestock associated *Culicoides* species, under both laboratory and field conditions.

**Methods:**

Batches of 500 field-collected *C. imicola* females were acclimatized at a predetermined range of temperatures (10–29 °C) and relative humidity (34–85%). After acclimatization, these females, prompted by a light source, were allowed to escape through a transparent plastic funnel into a paper cup, where they were counted after an hour. Flight activity under field conditions was determined seasonally by hourly light trap collections done overnight at four sites near cattle.

**Results:**

Experiments conducted at various test conditions in the laboratory indicated that flight activity started at 13 °C. Peak in activity was observed between 16 °C to 18 °C, and temperatures above 20 °C seemingly inhibit flight. Under field conditions, a peak in numbers collected was observed immediately after sunset. With mean nocturnal temperatures below 19 °C, more than 74% of the *Culicoides* were collected within two to three hours after sunset. With mean nocturnal temperature above 19 °C, the peak in numbers at sunset was sustained until after midnight, with somewhat higher numbers collected after midnight once temperatures dropped below 20 °C. No peak in numbers was observed at dawn. Although very low numbers were collected during the day, which partly may have been a result of the collecting method, *Culicoides* were present throughout periods of 24 hours. Humidity seemed to play a minor role in the regulation of flight activity.

**Conclusions:**

Abundance and species diversity results as obtained in this study indicated a high level of risk of virus transmission in the first hours following sunset. A strong relationship was found between host-seeking activity, and hence trap efficiency, and within limits, temperature. Light traps primarily measure flight activity and may as such underestimate adult abundance of *C. imicola* if deployed at temperatures outside thresholds of 16–20 °C.

## Background

Small blood-feeding midges in the genus *Culicoides* Latreille (Diptera: Ceratopogonidae) are the biological vectors of various pathogens that can cause a number of economically important diseases in livestock globally [1]. Of the more than 75 viruses associated with the 1368 described species of *Culicoides* worldwide [2], in excess of 23 have been isolated from the Imicola complex in the subgenus *Avaritia* Fox [3]. Based on their high abundance at livestock and confirmed oral susceptibility, *Culicoides imicola* Kieffer is considered the principal vector of bluetongue-, African horse sickness-, epizootic haemorrhagic disease- and equine enchephalosis viruses in South Africa [3–6]. It was recently reported that the susceptibility of *C. imicola* to Schmallenberg virus (Orthobunyavirus) could exceed that of the abundant and wide spread *Culicoides obsoletus* (Meigen) in Spain [7].

In addition to its susceptibility to a wide range of viruses of veterinary importance, *C. imicola* is one of the most widely distributed livestock-associated *Culicoides* species in the world. It has been recorded across the Afrotropical, Saharo-Arabian and Oriental regions in addition to areas of the Mediterranean basin [3]. It was moreover suggested that its geographical distribution could potentially extend further west in Europe than what is currently reported [8]. The geographical distribution and abundance of *Culicoides* species are, in addition to the availability of suitable hosts, regulated by weather conditions such as temperature, humidity and rainfall [1].

Blood-feeding success and associated risk of pathogen transmission by *Culicoides* species depends on their ability to be active when appropriate hosts are accessible. For the majority of *Culicoides* species, peak flight activity concurs with dusk and/or dawn [9, 10]. This peak may shift towards a diurnal pattern in cooler or a nocturnal pattern in hotter weather [11]. As observed in several species, diurnal activity is, however, not extraordinary [12–15]. Flight activity, like geographical distribution, depends on environmental factors such as temperature, humidity, wind and rainfall [16].

Insight into the circadian host-seeking flight activity of vectors, and the factors that regulate this, will be of importance to assess the risk of transmission of *Culicoides*-borne pathogens and the implementation of appropriate controls at farm level. Despite their proven role as vectors of a number of pathogens, little is known of the host-seeking activity of *Culicoides* species, including *C. imicola*, in southern Africa.

Despite limitations, various models of suction light traps have become the most popular and widely used tool to monitor *Culicoides* geographical distribution and abundance [17, 18]. Flight activity and hence trap efficiency are, amongst other factors, influenced by climatic factors such as temperature, humidity and wind speed [10, 19, 20]. Since insects may respond directly to wind, rain, humidity, temperature and light intensity, weather can be of particular importance. These factors can differ daily over relatively short geographical distances. In the present study, the influence of temperature and humidity on the flight activity of *C. imicola* under both laboratory and field conditions were determined.

## Methods

### Study site

Field collections of *Culicoides* were conducted at the Agricultural Research Council - Onderstepoort Veterinary Research (-25.6501, 28.1870; 1219 m above sea level) in South Africa. Mean annual rainfall ranges between 430–1017 mm with a peak in summer between November and March [21]. The annual mean daily maximum and minimum temperatures are 26.3 °C and 9.3 °C, respectively [21]. Onderstepoort 220 V ultraviolet light traps were operated overnight underneath the eaves of open sided and semi-closed stables housing between 20 and 40 cattle each. The traps were installed between 1.9 and 2 m above the ground. During the day, some cattle were out in open pens with concrete flooring adjoining the stables. Traps were 60 to 70 m apart and not in direct sight of each other. Trees and kikuyu lawns, varying in size, surrounded the stables. Vervet monkeys (*Chlorocebus pygerythrus*), wild birds and small rodents of various species were present at all of the sites.

### Flight behaviour under laboratory conditions

*Culicoides* were collected alive with light traps [22] at three to four sites as defined above during March and April 2017. The collected *Culicoides* were transferred into a black plastic box (450 × 330 × 280 mm) with a ventilated lid and allowed to exit the box through a white translucent funnel, mounted on one side of the box, into a 300 ml paper cup [22]. After 5% sucrose feeding on cotton wool pledgets at room temperature, pools of 500 *C. imicola* females were sorted on a refrigerated chill table. These pools were transferred to black plastic boxes (900 × 10 × 130 mm) through a 10 mm opening in the lid. A white transparent plastic funnel was mounted over a 120 mm opening on one side of the box. One hour before transfer of the midges, the boxes were placed inside climate controlled growth chambers, operated at one of seven set temperatures between 10–29 °C. After 15 to 20 min acclimatization the females were, prompted by a white fluorescent light source, allowed to escape through the funnel into a 300 ml white paper cup. After 60 min, *Culicoides* in the cups were immobilized for 30–50 s at -20 °C, and counted.

In the first series of experiments, the relative humidity in the chambers was not regulated. In subsequent trials, the humidity was kept at either 40%, 60% or 80%. Between six and ten replicates of 500 females each were conducted at the various temperature and humidity conditions. Temperature and humidity inside the holding box were recorded every 5 min with a DS1923-F5# Hygrochron iButton data logger (Fairbridge Technologies, Sandton, South Africa).

### Flight behaviour in the field

In the first field trial, on the 21th of April, conducted from 15:00 h until 9:00 h the next morning, light traps were operated at four sites as defined above. Insects were collected into distilled water to which 0.5% Savlon® (Johnson & Johnson, East London, South Africa) (clorhexidine gluconate 0.3 g/100 ml and cetrimide 3.0 g/100 ml) antiseptic had been added. The collection beakers were exchanged hourly and the collected insects stored in 80% ethanol at room temperature. All collected *Culicoides* were morphologically identified to species level under a stereomicroscope using a wing picture atlas of Afrotropical *Culicoides* [23]. Based on abdominal pigmentation [24], all females were age-graded into younger nulliparous (unpigmented), older parous (pigmented), gravid or freshly blood-fed. Captured males were also counted.

The continual presence of *Culicoides*, albeit in small numbers, in the traps before sunset and after sunrise in April prompted subsequent collections to be made over periods of 24 hours. In these trials, representative of different climatic conditions, on 7 July, 1 November and 17 February, hourly collections were made from 14:00 h on the first to 14:00 h the following day. Trials were restricted to nights with no wind and/or rain and temperature and humidity were monitored every 5 min with iButton data loggers.

### Statistical analysis

Analysis of variance (ANOVA) was used to analyse the data and because of unequal replications t probabilities of pairwise differences were used to compare the mean numbers of *C. imicola* females emerging from the holding boxes under the various temperature and humidity conditions. Data were analysed using the statistical program GenStat® [25] at the 5% level of significance.

Although the total numbers of *Culicoides* collected varied between the four sites, abundance patterns over time were similar. To compensate for potential site variation, the mean numbers collected hourly at each of the four sites were grouped. Hourly mean numbers of *C. imicola* collected were compared to the hourly mean temperature and relative humidity as measured from after sunset to before sunrise.

## Results

### Flight behaviour in the laboratory

In the first trial, six to nine replicates were conducted at each of seven temperatures between 10–29 °C (Table 1). The mean temperatures inside the holding boxes were to some extent lower than the set temperature of the chamber (Table 1). The mean number of midges to emerge from the holding boxes at the various temperatures differ significantly from each other as indicated by ANOVA (*F*_(6,38)_ = 16.51, *P* < 0.001). Subsequent t-tests for all pairwise differences of means indicated that the mean number of *Culicoides*, 139.8 ± 40.03, to emerge from the boxes at 17 °C (17.3 ± 0.74 °C) was significantly higher than that at any of the other temperatures (Table 1). At 10 °C (9.1 ± 0.24 °C), only two, of a potential 3000 *Culicoides*, emerged from the holding boxes. The low mean numbers, 0.3 ± 0.82, to emerge at 10 °C did not differ significantly from those at 13 °C (2.7 ± 3.20) (Table 1). The mean numbers of females to escape at 24 °C (57.7 ± 25.99) and 29 °C (55.2 ± 24.21) did not differ significantly. Emergence at 24 °C and 29 °C was significantly lower than that at 17 °C (Table 1). The relative humidity in the boxes, as influenced by the temperature, varied from 68.0 ± 2.6% at 10 °C to 34.3 ± 0.25% at 29 °C (Table 1).

**Table 1:** Mean numbers and standard deviation (SD) of field-collected *Culicoides imicola* females to emerge over a period of 60 minutes from holding boxes kept at various temperature and humidity levels

Set T (°C)	Mean T (SD)	Mean RH (SD)	No. of replicates (no of individuals)^*^	Total no. emerged (%)	Mean (SD)
10	9.1 (0.24)	68.0 (2.6)	6 (3000)	2 (0.02)	0.3 (0.82)^a^
13	12.3 (0.20)	53.5 (0.5)	6 (3000)	16 (0.5)	2.7 (3.20)^a^
15	14.5 (0.20)	51.9 (0.65)	6 (3000)	615 (20.5)	102.5 (64.46)^b^
17	17.3 (0.74)	49.7 (1.93)	9 (4500)	1258 (28.0)	139.8 (40.03)^c^
20	19.8 (0.58)	44.6 (0.61)	6 (3000)	415 (13.8)	69.2 (17.34)^b^
24	23.1 (0.47)	45.7 (0.52)	6 (3000)	346 (11.5)	57.7 (25.99)^d^
29	28.3 (0.07)	34.3 (0.25)	6 (3000)	331 (11.0)	55.2 (24.21)^d^

Numbers per row followed by a different letter were significantly different at the 5% level

*Abbreviations*: *RH* relative humidity, *T* temperature

^*^Each replicate consists of 500 individuals

To preclude the influence of humidity, the experiment, as set out above, was repeated at 60% RH. Although the humidity in the grow chamber was set at 60%, the mean humidity inside the holding boxes varied between 49.8 ± 11.22% at 29 °C and 62.3 ± 1.57% at 10 °C (Table 2). Similar to the previous trial the mean number of midges to emerge from the holding boxes at the various temperatures differ significantly (*F*_(5,44)_ = 20.81, *P* < 0.001). Subsequent t-tests for all pairwise differences of means indicated that the highest mean number of *Culicoides*, 135.1 ± 44.64, to emerge was, as in the previous trial, at 17 °C (16.9 ± 0.83 °C). The higher mean numbers to emergence at 17 °C (RH 57.5 ± 5.04%) was not significantly different from that of 130.6 ± 35.42 at 20 °C (RH 54.0 ± 6.99%) (Table 2). As in the first trial the lowest mean emergence, 2.3 ± 3.96, at 10 °C did not differ significantly from that of 19.1 ± 29.46 at 13 °C (Table 2). The mean numbers to emerge at 24 °C, 90.6 ± 45.06, and 29 °C, 75 ± 30.74, did not differ significantly. The emergence at both 24 °C and 29 °C was significantly lower than at 17 °C.

**Table 2:** Mean numbers and standard deviation (SD) of field-collected *Culicoides imicola* females to emerge over a period of 60 minutes from holding boxes kept at various temperature and 60% humidity level

Set T (°C)	Mean T (SD)	Mean RH (SD)	No. of replicates (no of individuals)^*^	Total no. emerged (%)	Mean (SD)
10	9.9 (0.85)	62.3 (1.57)	8 (4000)	18 (0.5)	2.3 (3.96)^a^
13	12.9 (1.08)	56.5 (2.78)	8 (4000)	153 (3.8)	19.1 (29.46)^a^
17	16.9 (0.83)	57.5 (5.04)	8 (4000)	1081 (27.0)	135.1 (44.64)^b^
20	19.8 (0.89)	54.0 (6.99)	8 (4000)	1045 (26.1)	130.6 (35.42)^b^
24	23.9 (1.06)	53.5 (9.11)	10 (5000)	906 (18.1)	90.6 (45.06)^c^
29	28.9 (0.80)	49.8 (11.22)	8 (4000)	600 (15.0)	75 (30.74)^c^

Numbers per row followed by a different letter were significantly different at the 5% level

*Abbreviations*: *RH* relative humidity, *T* temperature

^*^Each replicate consists of 500 individuals

To substantiate the influence of humidity on flight activity, the results obtained at various humidity levels at 17 °C were compared (Table 3). In this trial, the mean humidity inside the boxes ranged from 46.4 ± 2.1% to 84.9 ± 2.12% and the temperature varied between 16.5 ± 0.45 °C and 17.7 ± 0.37 °C (Table 3). The mean numbers of *Culicoides* to escape from the holding boxes, ranged from 135.1 ± 44.64 at 60% RH to 178.8 ± 44.62 at 80% RH and were not significantly different (*F*_(3,29)_ = 2.51, *P* = 0.078) at the various humidity levels.

**Table 3:** Mean numbers and standard deviation (SD) of field-collected *Culicoides imicola* females to emerge over a period of 60 minutes from holding boxes kept at 17 °C at various humidity levels

Mean T (SD)	Set RH	Mean RH (SD)	No. of replicates (no of individuals)^a^	Total no. emerged (%)	Mean (SD)
17.3 (0.74)	not set	49.7 (1.93)	9 (4500)	1258 (28.0)	139.8 (40.03)
16.5 (0.45)	40	46.4 (2.1)	8 (4000)	1430 (35.8)	178.8 (44.62)
16.9 (0.83)	60	57.5 (5.04)	8 (4000)	1081 (27.0)	135.1 (44.64)
17.7 (0.37)	80	84.9 (2.12)	8 (4000)	1381 (34.5)	172.6 (30.09)

*Abbreviations*: *RH* relative humidity, *T* temperature

^a^Each replicate consists of 500 individuals

**Fig. 1:**
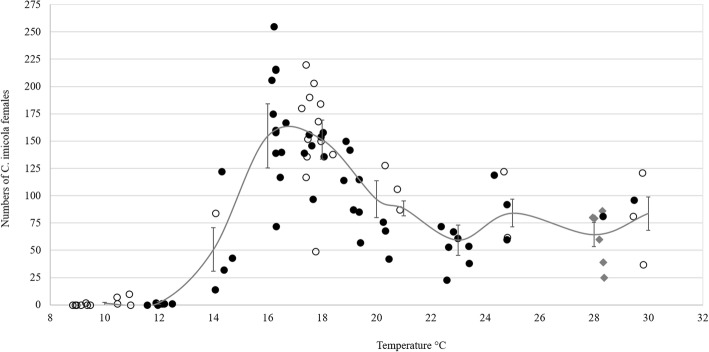
Mean numbers and standard deviation of *Culicoides imicola* females to emerge from holding boxes kept at various temperature and humidity levels. Open circles: humidity > 60%; closed circles: humidity 40–60%; diamonds: humidity < 40%

An aggregated comparison of the data (Fig. 1) confirmed low emergence at 10 °C and indicated that activity, independent of humidity, only started to increase once temperatures rose above 13 °C. Figure 1 shows that peak emergence was recorded between 16–18 °C and that it started to decrease when temperatures increased above 19–20 °C. There were no significant differences in the numbers to emerge from the holding boxes once the temperature was above 20 °C (up to 29 °C) (Fig. 1). No correlation between the mean emergence and RH, ranging between 46.4–84.9%, could be established (Fig. 1). Humidity seemed to play a minor role in the regulation of flight activity in the present set of experiments.

### Flight behaviour under field conditions

#### April 2017

**Fig. 2:**
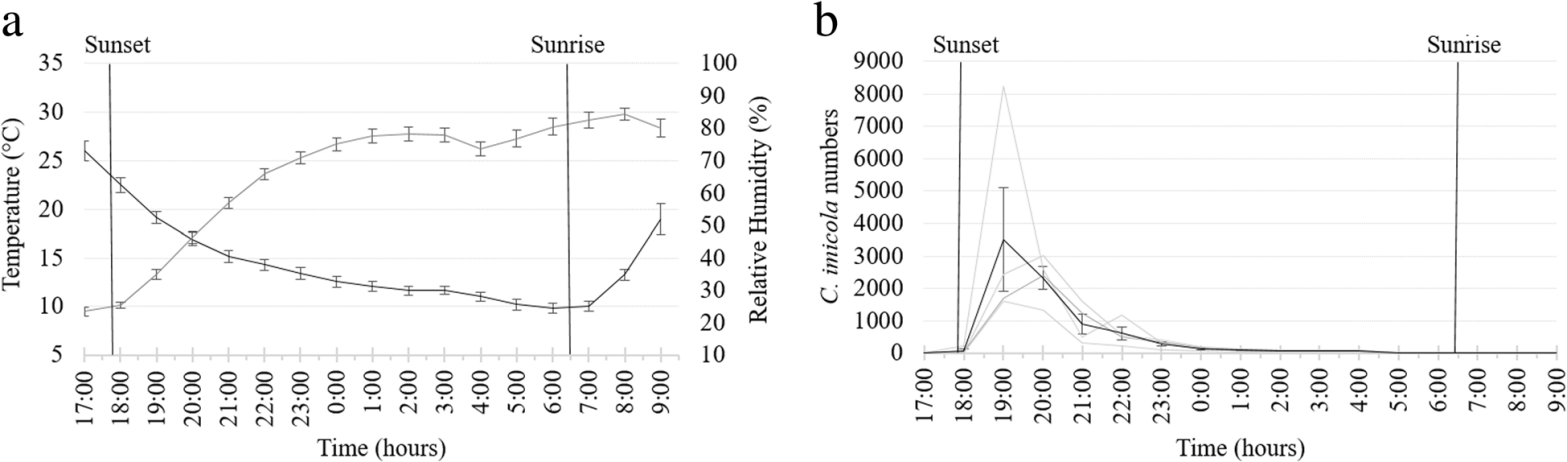
Mean and standard deviation in hourly temperature (black line) and relative humidity (grey line) (**a**) and *Culicoides imicola* numbers (black line) (**b**) as determined in 68 light trap collections made at four sites near cattle at the ARC-OVR from 17:00 h on 21 April to 9:00 h on 22 April 2017. The *C. imicola* numbers collected at the four individual sites are indicated with grey lines. Vertical black lines indicate sunset and sunrise

During the first trial, collections were conducted from 17:00 h on 21 April to 9:00 h on 22 April, and 17 one hourly collections were made at each of four sites. Sunset occurred at 17:45 h on 21 April and sunrise at 6:26 h the following day [26]. The hourly mean overall temperature, 14.6 °C, dropped from 26.0 ± 2.04 °C at 17:00 h to 9.8 ± 1.08 °C at 6:00 h (Fig. 2a). Inversely, the mean overall relative humidity, 65.4%, increased from 23.4 ± 2.90% at 17:00 h to 84.7 ± 3.46% at 8:00 h.

*Culicoides imicola* accounted for 99.3% (32,577) of 32,806 *Culicoides* collected in the 68 hourly collections made at the four sites. The total numbers of *C. imicola* collected at individual sites varied between 3740 (99.2%) and 13,615 (99.4%). Although the mean numbers collected varied between sites it peaked dramatically immediately after sunset at all four sites with 83% (26,920 of 32,577) of all *Culicoides* being collected within the first two hours after sunset (Fig. 2b). During the two hours of peak abundance, 19:00 h to 21:00 h, the mean temperature decreased from 19.1 ± 1.17 °C to 17.0 ± 1.29 °C.

The gradual decrease in the mean hourly temperature (13.9 °C), from 19.1 ± 1.17 °C at 19:00 h to 9.8 ± 1.07 °C at 6:00 h between sunset and sunrise correlated strongly (*r*_(10)_ = 0.93, *P* < 0.0001) with the decrease in the mean numbers of *C. imicola*. Inversely, the mean relative humidity, increasing from 46.4 ± 3.51% to 82.6 ± 4.77%, showed a similar negative correlation (*r*_(10)_ = -0.97, *P* < 0.0001).

**Fig. 3:**
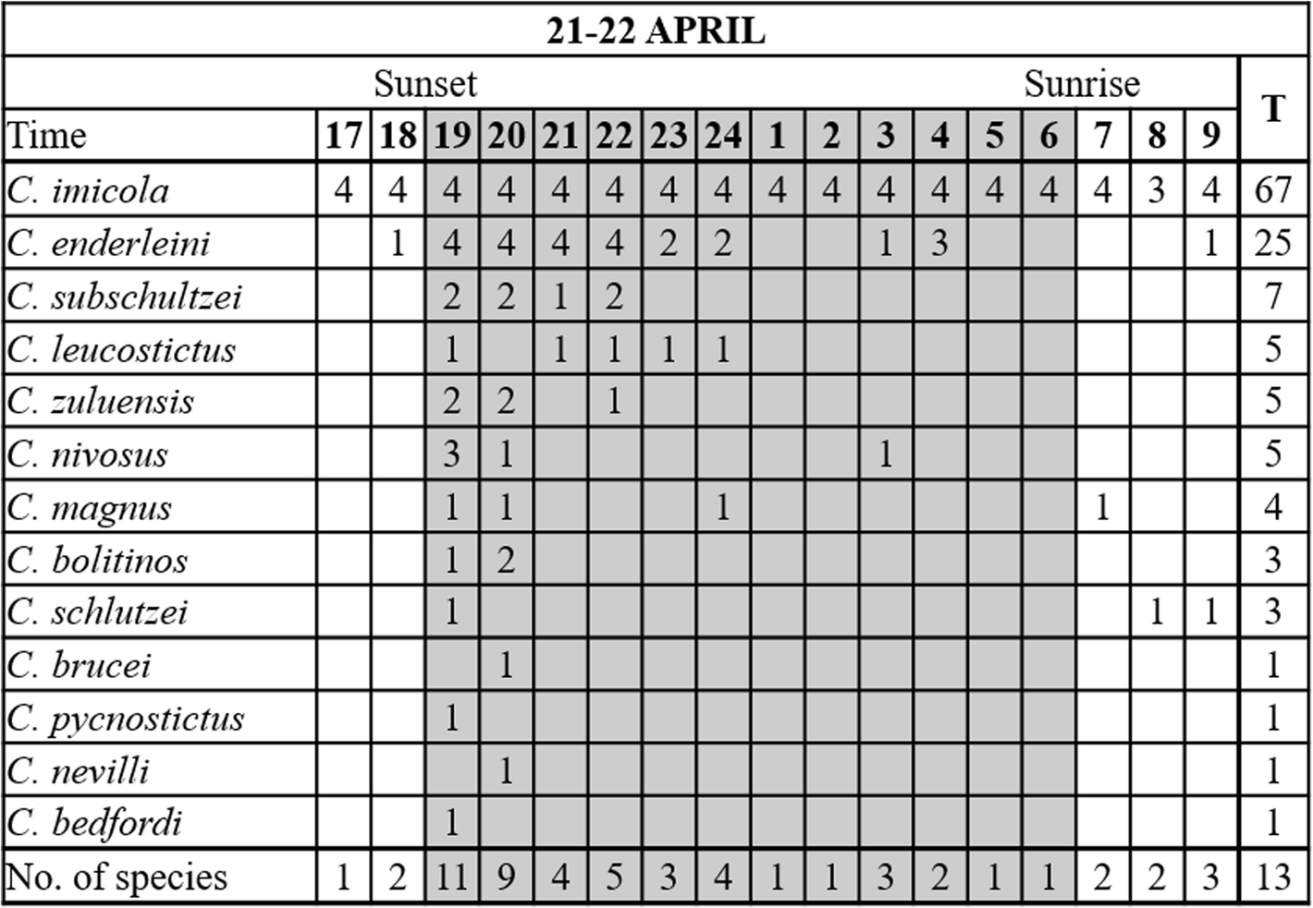
Hourly species richness of a *Culicoides* population as determined with 68 light trap collections at four sites near cattle at the ARC-OVR from 17:00 h on 21 April to 9:00 h on 22 April 2017. The numbers (1–4) indicate the number of sites at which the species was present. *Abbreviation*: T, the total number of collections in which the species was encountered

The mean temperature before sunrise was 9.8 ± 1.08 °C and no peak in *Culicoides* numbers was observed before, during or after sunrise (Fig. 2b). Although only low numbers, less than 10 *Culicoides* per trap, were collected before sunset and after sunrise, *Culicoides* were present at one or more of the sites throughout the 17-h period (Fig. 3).

*Culicoides* belonging to at least 13 species were collected (Fig. 3). The dominant species, *C. imicola*, was present in 67 of the 68 hourly collections. Similar to abundance, species richness was the highest during initial two hours after sunset and all species eventually collected were encountered between 19:00 h and 20:00 h (Fig. 3). Species collected before sunset and after sunrise included low numbers of *C. imicola*, *Culicoides enderleini* Cornet & Brunhes, *Culicoides magnus* Colaco and *Culicoides schultzei* (Enderlein) (Fig. 3).

Nulliparous *C. imicola* females, representing 71.5% of the *C. imicola* collected*,* was the dominant physiological grouping at all four sites throughout the collection period. Similarly parous females, representing 27.3% of all *C. imicola* collected*,* were present throughout this period. Although the parous rates differed between the sites, it did not differ over time. Freshly blood-engorged *C. imicola* females, representing 0.3% of the *Culicoides*, were only present between 18:00 h and 1:00 h and males, representing 0.9%, from 19:00 h to 9:00 h.

#### July 2017

**Fig. 4:**
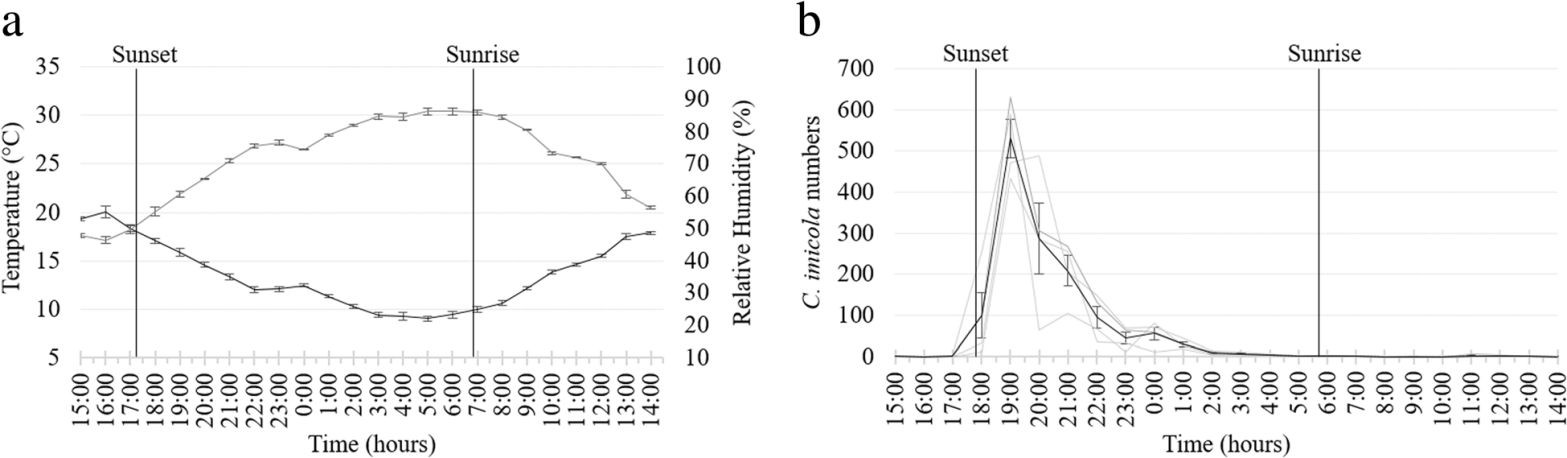
Mean and standard deviation in hourly temperature (black line) and relative humidity (grey line) (**a**) and *Culicoides imicola* numbers (black line) (**b**) as determined in 96 light trap collections at four sites near cattle at the ARC-OVR from 15:00 h on 7 July to 14:00 h on 8 July 2017. The *C. imicola* numbers collected made at the four individual sites are indicated with grey lines. Vertical black lines indicate sunset and sunrise

During the second trial, 96 hourly collections were made at the same four sites as in April from 14:00 h on 7 July to 14:00 h on 8 July. Sunset was at 17:29 h and sunrise at 6:55 h the following day [26]. The hourly overall mean temperature, 13.6 °C, ranged from 20.0 ± 1.19 °C at 16:00 h to 9.0 ± 0.50 °C at 5:00 h and the mean overall relative humidity, 71.3%, from 46.4 ± 2.34% at 16:00 h to 86.2 ± 1.89% at 6:00 h (Fig. 4a).

Despite the fact that collections were made over 24 hours, the total number of *Culicoides* collected in July (5540) was lower than that in April (32,806). *Culicoides imicola*, representing 5526 (99.7%) of 5540 *Culicoides* collected, was once again the dominant species. The total number of *C. imicola* collected varied between 1098 (99.7%) and 1632 (99.9%) at the sites.

**Fig. 5:**
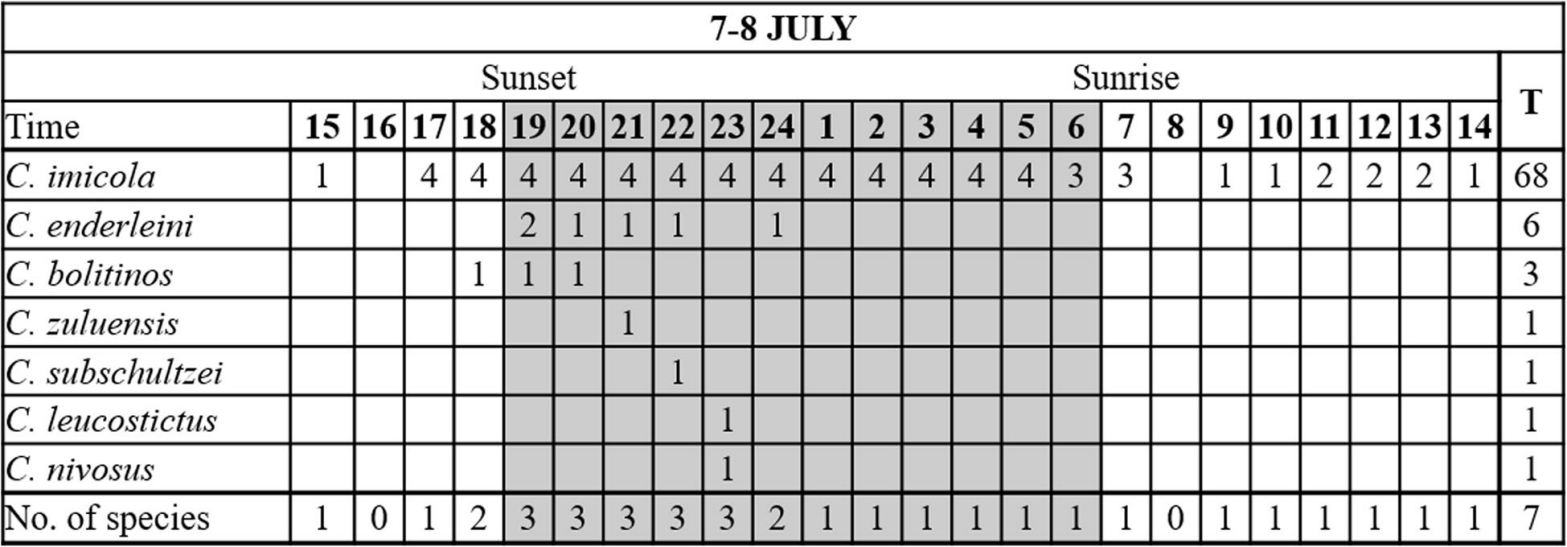
Hourly species richness of a *Culicoides* population as determined with 96 light trap collections at four sites near cattle at the ARC-OVR from 15:00 h on 7 July to 14:00 h on 8 July 2017. The numbers (1–4) indicate the number of sites at which the species was present. *Abbreviation*: T, the total number of hourly collections in which the species was encountered

As in April, the numbers of *Culicoides* collected peaked immediately after sunset with 4115 (74%) of 5540 *Culicoides* collected within the first three hours, 18:00 h to 21:00 h, after sunset at all four sites (Fig. 4b). During this period, the mean temperature decreased from 15.9 ± 0.73 °C at 18:00 h to 13.3 ± 0.64 °C at 20:00 h (Fig. 4a). Between sunset and sunrise the mean temperature, 11.6 °C, dropped from 15.9 ± 0.73 °C at 18:00 h to 9.5 ± 0.72 °C at 5:00 h. The decrease in mean numbers of *C. imicola* collected between sunset and sunrise correlated significantly with the mean hourly temperature (*r*_(10)_ = 0.90, *P* < 0.0001) and relative humidity (*r*_(10)_ = -0.92, *P* < 0.0001). The mean temperature at sunrise was 9.8 ± 1.08 °C with no observable peak in numbers collected (Fig. 4a, b). Though relatively low mean numbers were collected before sunset (0.5) and after sunrise (0.7), *C. imicola* was present at one or more of the four sites between 7:00 h and 14:00 h (Fig. 5).

Even though the mean temperature fell below 10 °C for three or more consecutive hours after midnight (Fig. 4a) during July, low numbers of *C. imicola* were still present in most of the collections (Fig. 4b).

With only seven *Culicoides* species collected species richness was lower in July compared to that in April. The dominant species, *C. imicola*, was present in 68 of the 96 hourly collections (Fig. 5). All species eventually found were collected before midnight. Species collected before sunset were *C. imicola* and *Culicoides bolitinos* Meiswinkel. *Culicoides imicola* was the only species to be collected after sunrise (Fig. 5).

Nulliparous *C. imicola* females, representing 64.0% of those collected, was the dominant physiological grouping. The mean parous rate, 34.5%, was higher than that in April and although it varied between sites, it did stay constant during the period. Blood engorged *C. imicola* females, accounting for 0.4% of the collection, were present between 17:00 h and 22:00 h and males, accounting for 0.9%, between 10:00 h and 23:00 h.

#### November 2017

**Fig. 6:**
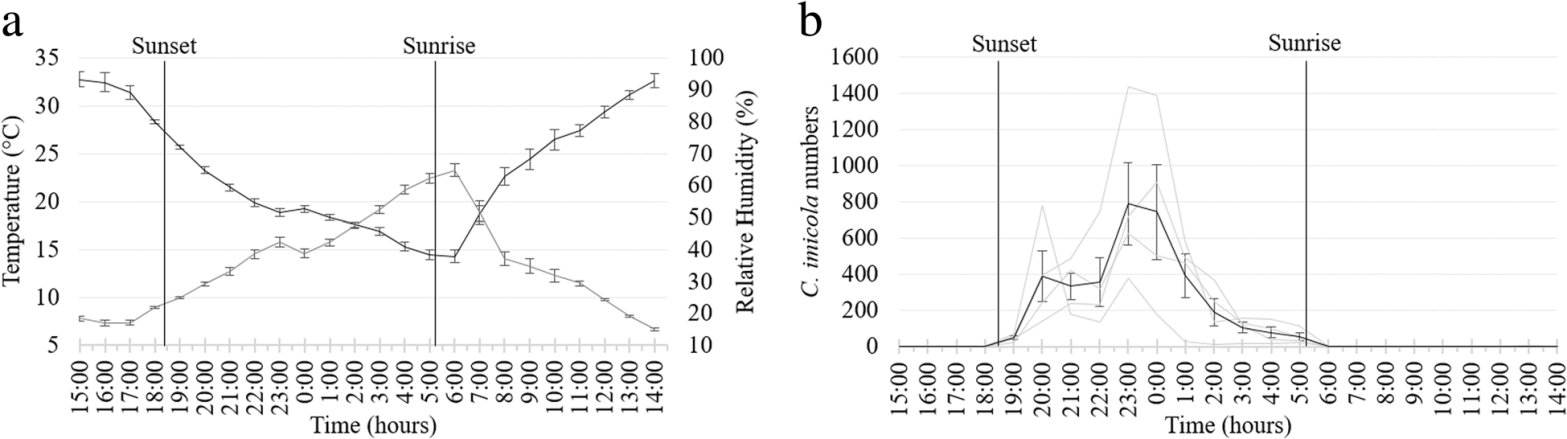
Mean and standard deviation in hourly temperature (black line) and relative humidity (grey line) (**a**) and *Culicoides imicola* numbers (black line) (**b**) as determined with 96 light trap collections at four sites near cattle at the ARC-OVR from 15:00 h on 1 November to 14:00 h on 2 November 2017. The *C. imicola* numbers collected at the four individual sites are indicated with grey lines. Vertical black lines indicate sunset and sunrise

During the third survey, sunset occurred at 18:23 h on 1 November and sunrise at 5:17 h the following morning [26]. The mean temperature, 23.5 °C, ranging from 32.8 ± 1.57 °C at 15:00 h to 14.3 ± 1.28 °C at 6:00 h, was higher than that in the previous two trials (Fig. 6a).

As in the previous trials, *C. imicola* dominated. This species represented 96.3% (13,972) of 14,504 *Culicoides* collected in 96 hourly collections made at all of the four sites from 14:00 h on 1 November to 14:00 h on 2 November. Its proportional representation at the four sites varied between 94.1% and 97.5%. As in April and July, the numbers collected increased sharply directly after sunset (Fig. 6b). The mean temperature, 19.2 °C, decreased from 25.7 ± 0.39 °C at 19:00 h (after sunset) to 14.3 ± 1.28 °C at 5:00 h (before sunrise) (Fig. 6a). Contrary to the previous two trials, the numbers collected after sunset did not decrease within the first three hours after sunset, and the highest mean numbers were found later, between 23:00 h and midnight, once the mean temperature dropped below 20 °C (Fig. 6a). The overall correlation between mean numbers collected from after sunset to before sunrise and temperature was low (*r*_(8)_ = 0.51, *P* = 0.128). After midnight the mean numbers started to decrease and between 23:00 h and 5:00 h, with mean temperatures decreasing from 18.9 ± 0.85 °C to 14.5 ± 0.97 °C, the correlation (*r*_(5)_ = 0.86, *P* = 0.014) between temperature and numbers collected increased. In November, the variation in the numbers collected hourly at each of the four sites was more pronounced than in the previous surveys (Fig. 6b). The mean temperature at sunrise was 14.5 ± 0.97 °C and no peak in numbers was observed.

**Fig. 7:**
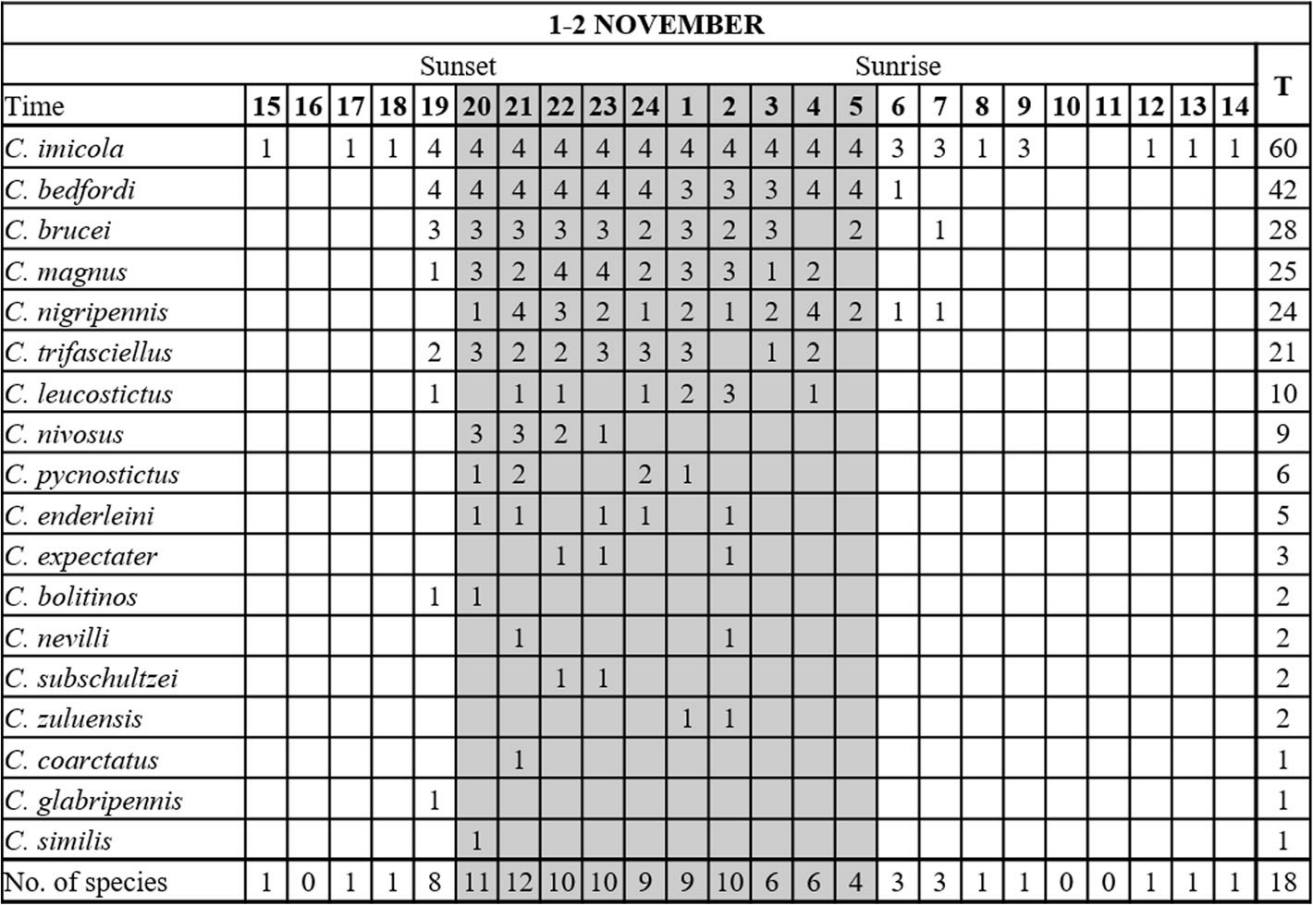
Hourly species richness of a *Culicoides* population as determined with 96 light trap collections at four sites near cattle at the ARC-OVR in from 15:00 h on 1 November to 14:00 h on 2 November 2018. The numbers (1–4) indicate the number of sites at which the species was present. *Abbreviation*: T, the total number of hourly collections in which the species was encountered

With at least 18 species collected species richness was higher than in the first two trials. Seven of these 18 species were present throughout the night (Fig. 7). Except for *Culicoides zuluensis* de Meillon, all species were found before midnight. Low numbers of *C. imicola* were collected at one site before sunset (Fig. 6b). Species collected up to two hours after sunrise included *C. imicola*, *Culicoides brucei* Austen and members of the Nigripennis group.

The mean proportional representation, 54.2%, of nulliparous *C. imicola* females, ranging from 51.1% to 60.9% between sites, was lower than in the prior trials. The proportion of parous females ranged from 34.2% to 45.7% between sites and although it differed between sites, it did not differ over time. Blood engorged females, at 1.4% of the total collected, were present from 19:00 h until at least 4:00 h at one or more of the sites. Gravid females, making up 0.2% of the total numbers, were present from 20:00 h to 3:00 h and males, comprising 1.6%, were absent from 6:00 h to 11:00 h.

#### February 2018

**Fig. 8:**
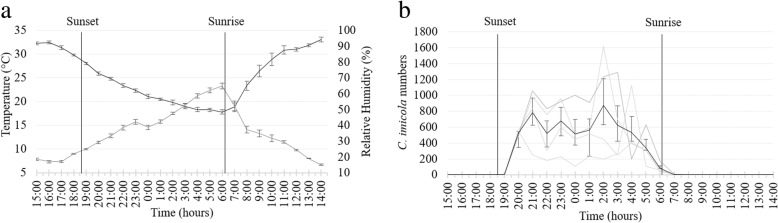
Mean and standard deviation in hourly temperature (black line) and relative humidity (grey line) (**a**) and *Culicoides imicola* numbers (black line) (**b**) as determined with 96 light trap collections at four sites near cattle at the ARC-OVR from 15:00 h on 16 February to 14:00 h on 8 February 2018. The *C. imicola* numbers collected at the four individual sites are indicated with grey lines. Vertical black lines indicate sunset and sunrise

In the fourth trial, 24 hourly collections were made at each of the four sites from 14:00 h on 16 to 14:00 h on 17 February. Sunset was at 18:48 h and sunrise at 5:53 h the following day [26]. The mean temperature, 25.4 °C, over the entire collection period ranged from 17.8 ± 0.92 °C at 6:00 h to 32.00 ± 1.12 °C at 14:00 h and the mean humidity from 26.2 ± 1.13% at 16:00 h to 81.0 ± 3.94% at 6:00 h (Fig. 8a).

During this trial, 25,198 *Culicoides* were collected in 96 hourly collections at the four sites. With a mean abundance of 96.3%, the proportional representation of *C. imicola* ranged from 91.7% to 98.0% at the four sites. As in all three previous surveys, the numbers collected increased after sunset (Fig. 8b). The mean temperature at 20:00 h (after sunset) was 25.90 ± 0.70 °C and, contrary to the previous trials, the peak in mean numbers collected was not directly after sunset but at least an hour later at 21:00 h (Fig. 8b). As in November, this peak did not decline within two to three hours and relatively high numbers were collected throughout the night (Fig. 8b). The highest mean numbers were once again collected once the mean temperature dropped below 20 °C at 2:00 h (Fig. 8b). After sunset, the mean temperature decreased gradually from 25.9 °C to a minimum of 18.3 ± 0.71 °C before sunrise. Between 20:00 h (after sunset) and 6:00 h (before sunrise) the overall correlation between the mean numbers collected hourly and temperature was low (*r*_(9)_ = 0.42, *P* = 0.204). Between 2:00 h, once temperatures fell below 20 °C, and 6:00 h this correlation increased (*r*_(3)_ = 0.95, *P* = 0.014). The variation in the numbers collected hourly at each of the sites was, similar to the survey in November, greater than that in April and July.

**Fig. 9:**
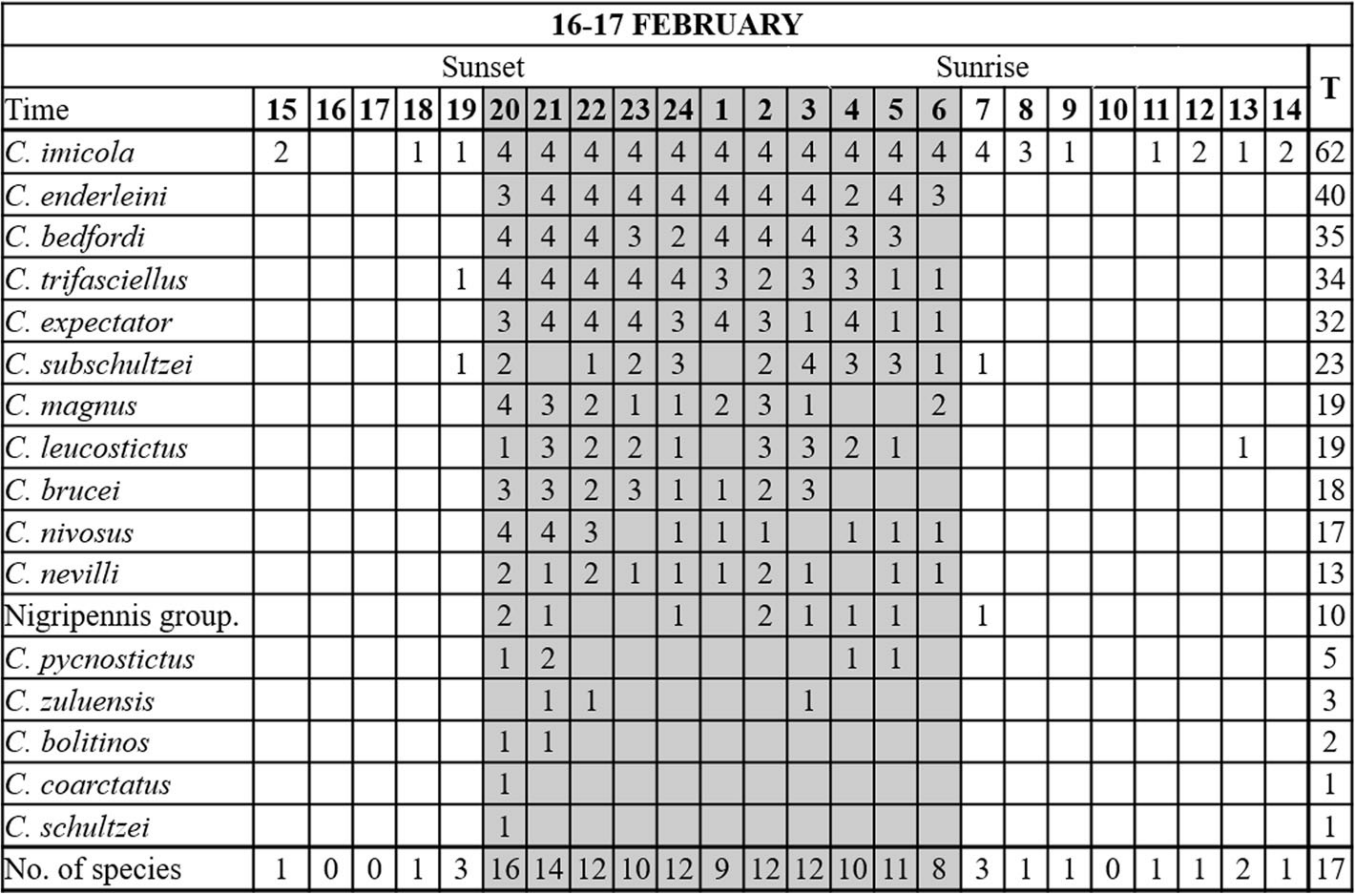
Hourly species richness of a *Culicoides* population as determined with 96 light trap collections at four sites near cattle at the ARC-OVR from 15:00 h on 16 February to 14:00 h on 17 February 2018. The numbers (1–4) indicate the number of sites at which the species was present. *Abbreviation*: T, the total number of hourly collections in which the species was encountered

With 17 species collected, species richness was, as in November, relatively high (Fig. 9). Although species richness was the highest immediately after sunset, most species were present throughout the night. *Culicoides imicola* was only absent in seven of 96 collections made. Other species collected before sunset or after sunrise included *Culicoides trifasciellus* Goetghebuer, *Culicoides subschultzei* Cornet & Brunhes, *Culicoides leucostictus* Kieffer and members of the Nigripennis group (Fig. 9).

The dominant physiological grouping was once again nulliparous *C. imicola* females. The mean proportional representation, 57.3%, ranging from 52.5% to 59.7% between sites, was similar to that in November. The proportion of parous females ranged between 30.8–36.4% and although differing between sites, did not alter over time. Gravid and blood-engorged females, comprising less than 1% of the totals, were present at all four sites. Contrary to the previous surveys, relatively high proportions, 16.1% to 3.3%, of *C. imicola* males were found. Males were present throughout the night.

## Discussion

Studies under controlled conditions revealed low flight activity for *C. imicola* females below 10 °C, with activity independent of relative humidity and only starting to increase once temperatures rose above 13 °C. In the laboratory, optimum flight activity, as reflected from the number of individuals moving towards a white light source, was recorded between 16 °C and 18 °C. Flight activity was seemingly supressed at temperatures above 19–20 °C. Flight activity also did not differ significantly between 20 °C and 29 °C. Following a similar protocol low flight activity below 10 °C, with an increase between 10 °C and 20 °C, was previously described for *Culicoides oxystoma* Kieffer and *Culicoides maculatus* (Shiraki) in Japan [27]. These temperatures, at which activity starts to increase, correspond with the minimum temperature of 11 °C to 13 °C, at which orbiviral replication commences in a number of species, including *C. imicola* [28].

The present results support presence/absence models for *C. imicola* [29, 30], based on a mean minimum temperature threshold of 12.5 °C [31, 32], and predictions that it would be present in areas with mean annual temperature between 12–20 °C [33]. Under laboratory conditions, no correlation was found between relative humidity and flight activity. Humidity, although correlated with temperature in the field, seems to play a secondary role in flight initiation.

Despite low flight activity recorded in the laboratory, *Culicoides*, including *C. imicola*, was found in traps operated near cattle at environmental temperatures below 10 °C in July. This indicates that factors such as attraction cues from the host linked to the hunger status of the female might also play a role in flight initiation. The involvement of additional factors, besides temperature, in flight initiation could partly explain the relatively low escape rates (< 50%) and migration to the light source under laboratory conditions in the present study. The field-collected females were fed on a 5% sucrose solution before evaluation and once inside the holding boxes, exposure to environmental cues that could have initiated flight would have been minimal [34]. Due to greater attractiveness [35], an ultraviolet light source in the growth chambers may have proportionally increased the number of midges emerging from the holding boxes at the various temperatures. The influence of light source, if any, on the temperature at which flight activity are initiated and maintained still need to be determined.

The field trials indicated that flight activity in *C. imicola*, as measured with light traps, peaked markedly directly after sunset. This trait has been described for several *Culicoides* species in America [11], Europe [10, 36], Australia [9, 20] as well as for *C. imicola* in Israel [37] and Senegal [38, 39]. This peak in numbers at sunset was, however, delayed if the temperature at sunset was above 25 °C as seen in February.

During April and July, with mean nocturnal temperatures below 19 °C, between 74–83% of the *Culicoides* were collected within two to three hours after sunset. During these surveys, the peak in numbers at dusk decreased directly relative to the decrease in mean temperatures. A similar correlation between flight activity and temperature was observed for *C. obsoletus* in France [36]. In November and February, with the mean nocturnal temperature above 19 °C, the peak in numbers at sunset was prolonged until after midnight. A slight increase in numbers collected were observed once the mean temperature dropped below 20 °C after midnight. At similar temperatures in Kenya [40] and Israel [37], *C. imicola* was also present in relatively high numbers throughout the night. A similar peak in numbers in *C. imicola* (syn. *C. pallidipennis*), and members of the Schultzei group, was observed at midnight, with environmental temperatures around 20 °C, in Kenya [40]. Similar to the present study, it was reported that, in Kenya, the flight activity of both these species was correlated to an increase in temperature up to 20 °C and suppressed after sunset at temperatures above 25 °C [40].

Several authors claim that rapidly changing levels of light intensity initiate flight and provoke *Culicoides* attacks [41–44]. Bimodal flight activity (at dusk and dawn) has been recorded for a number of *Culicoides* species [9–11], including some Afrotropical ones [40, 42]. In some instances, the peak at dawn can be higher than that at dusk [37]. In the present study, with mean temperatures before sunrise, varying between 9.0 °C (July) and 17.8 °C (February), corresponding with the coldest part of the night, no peak in numbers was seen at dawn. This is in agreement with previous studies, conducted in the same area in February 2009, where a peak at dawn was also apparently absent [45]. Due to the collection method, it is possible that a peak after sunrise could have been overlooked. The relatively low numbers collected immediately before and during sunrise, however, signify that a significant peak may have been absent.

As with abundance, *Culicoides* species richness was at a maximum the first few hours after sunset in April and July. Although all species present were collected within the first six hours after sunset, species richness was high throughout the night in November and February.

Any variation in the response of *Culicoides* to lights and hosts is of importance, taking into account that several studies utilized light traps to determine flight activity as an indication of abundance, when it is actually the attack rate on the host that is of relevance [18, 46]. Despite inefficiencies of light traps in collecting diurnal species, low numbers of *C. imicola* were found throughout 24 hours in the present study. These results support previous South African studies indicating that host-seeking and blood-feeding commence before sunset on livestock [47] and wild animals [12]. The presence of freshly blood-engorged females in the traps during the day in the present study accentuates this observation. Several diurnally active species, e.g. *Culicoides paraensis* (Goeldi), *Culicoides actoni* Smith, *Culicoides chiopterus* (Meigen) have been recorded [13–15, 46]. *Culicoides chiopterus* can peak in abundance, as indicated by sweep netting, up to two hours before sunset and their abundance and potential role in virus transmission is underestimated in light trap collections [46]. Similarly it was found that the peak in *C. obsoletus* activity in Europe can shift from after sunset in summer to before sunset in spring and autumn [36]. Despite the presence of *Culicoides*, as determined using light traps, the stabling of horses during the day from before dusk until after dawn is still considered, and has been for over a century [48], as an effective method of preventing African horse sickness in South Africa. The epidemiological significance of daytime biting by *Culicoides* requires further study because of its implication for the diurnal transmission of viruses. It need to be emphasized that light trap results do not inevitably relates to the attack or biting rate on host animals [15, 47].

The unwavering dominance of non-blood engorged nulliparous and parous females in the light trap collections throughout the collection period reinforces the fact that this method is predisposed to groups with high host-seeking activity. Although this trait may increase monitoring sensitivity to establish relative risk for virus transmission, it will underestimate overall *Culicoides* abundance in an area.

The present field and laboratory data indicated a linear correlation between flight activity and temperatures between 10–20 °C. At mean nocturnal temperatures below 20 °C host-seeking flight activity, and feeding, will be condensed into the three hours immediately after sunset. Once mean nocturnal temperatures increase above 20 °C, host-seeking and flight activity, and the risk of virus transmission, will be extended over longer periods and not be restricted to a one- to three-hour period after sunset. This phenomenon was reinforced by an increase in variation between sites in November and February. At nocturnal temperatures above 20 °C, the movement of host animals in relation to the trap may play a more meaningful role in regulating localised abundance and trapping efficiency.

In evaluating *Culicoides* abundance, determined by light traps, it must be taken into consideration that host-seeking females represent a relatively small part of the entire population. Temperature not only influences flight activity but also significantly affects the development time of sub-adult stages, vector competence and adult survival. A significant decrease in egg batch size as well as in the number of days from blood-feeding to ovipositing and successive blood-feeding with increasing temperature was observed for *Culicoides sonorensis* Wirth & Jones as well as *C. imicola* [49, 50]. At 10 °C, the median oviposition time in *C. sonorensis* may be as long as 34 days as compared to 8–12 days at 30 °C [49]. Prolonged ovipositing time will reduce blood-feeding activity and may lead to lower flight activity. Shorter development time above 20 °C will lead to an increase in abundance. At these temperatures, blood digestion and virus replication in infected females will increase, resulting in a decrease in the period between blood meals and an increase in feeding frequency and potential virus transmission [28, 51–53]. These variables may not be reflected in flight activity and light trap results.

The present results need to be evaluated against the fact that the relationship between flight activity, abundance and light trap efficiency are not clearly defined [18]. The observation that environmental temperature influences flight activity, and hence light trap efficiency, adds to factors that contribute to the variability in light trap results and reliable data comparison between geographical areas. The number of factors that can influence light trap results underlined that light trap results need to be used with caution in the production of risk maps and models for *Culicoides* species and associated disease transmission.

## Conclusions

Based on abundance, as well as on species richness, the present study indicates favourable conditions to support a high level of flight and host-seeking activity, and an associated risk of potential virus transmission, in the first hours after sunset. The present study highlights a strong relationship between flight activity as an indicator of host-seeking activity, and, within limits, temperature. Optimal flight activity, and hence trap efficiency, may be limited to a relatively narrow band between 16–20 °C. Light traps primarily measure flight activity and may as such underestimate adult abundance of *C. imicola* if deployed at temperatures outside of these thresholds.
